# MamNet-PT: A Mamba-enhanced hybrid architecture with selective state-space modeling for uncertainty-aware brain tumor segmentation

**DOI:** 10.1371/journal.pone.0351667

**Published:** 2026-07-15

**Authors:** Yu Sun, Yihang Qin

**Affiliations:** 1 School of Special Education, Changchun University, Changchun, China; 2 School of Computer Science and Technology, Changchun University, Changchun, China; The University of Texas, MD Anderson Cancer Center, UNITED STATES OF AMERICA

## Abstract

Precise segmentation of brain tumors from MRI remains a challenging problem in medical image analysis because tumor regions exhibit substantial size variability, diffuse and infiltrative boundaries, and severe foreground-background imbalance. To address these challenges, we propose MamNet-PT, a hybrid segmentation architecture that integrates efficient long-range dependency modeling, multi-resolution feature aggregation, and uncertainty-aware prediction within a unified framework. First, a selective state-space model is embedded into the U-Net-based feature pathway to capture long-range spatial dependencies with linear computational complexity, which is particularly important for irregular and spatially extended tumor regions. Second, a pre-trained ResNet-50 encoder is used to improve feature robustness under limited annotated medical data. Third, a gated feature interaction mechanism adaptively balances Mamba-derived global contextual features and CNN-derived local boundary features, avoiding simple feature concatenation or uncontrolled module stacking. In addition, a multi-resolution pyramid fusion module strengthens scale-aware representation of small enhancing foci and extensive edema, while Monte Carlo Dropout-based uncertainty estimation provides spatial confidence maps for retrospective confidence characterization and failure-mode analysis. On the BraTS2020 benchmark, MamNet-PT achieves a Dice score of 96.7% and an Intersection over Union of 95.4%, outperforming representative CNN-Transformer and Mamba-based segmentation baselines. Ablation experiments further confirm that the performance gain is attributable to the complementary effects of selective state-space modeling, gated global-local fusion, multi-resolution aggregation, and uncertainty-aware inference. These results suggest that MamNet-PT is a promising research framework for accurate and efficient brain tumor segmentation under retrospective benchmark evaluation.

## 1. Introduction

Precise brain tumor segmentation requires a model that can integrate global anatomical context for spatially extended edema and infiltrative growth while preserving fine local evidence for small enhancing foci and irregular tumor margins.CNN-based models, including U-Net variants [1](Fine-grained local modeling capabilities of U-Net through synergistic integration [2]), preserve local texture and boundary cues, but their convolutional inductive bias and limited receptive fields make long-range dependency modeling difficult, which can fragment diffuse lesions or underestimate spatially extended tumor extent. Transformers, in contrast, provide stronger global modeling, yet their quadratic self-attention cost and high data demand are poorly matched to brain MRI segmentation [3], where annotations are scarce and overfitting is a persistent risk.

Recent advances have addressed parts of this challenge but not its core mechanism. State-space models [4] and Mamba-style architectures [5] offer an efficient alternative for long-range dependency modeling; The pre-trained visual backbone [6] transfers rich prior knowledge from natural images [7] to the model, thereby enhancing representation learning in scenarios with limited medical data; multi-scale fusion [8] helps capture tumors with large size variation; curriculum learning [9], Bayesian uncertainty estimation [10] and confidence calibration [11] provide mechanisms for quantifying predictive uncertainty, and multi-objective optimization [12] improve training stability, confidence awareness, and class-imbalance handling; and diversity-aware integration strategies [13] can strengthen robustness. However, these approaches are often introduced as parallel improvements rather than as a principled solution to the global-local conflict that defines brain tumor segmentation. As a result, prior work still struggles with the diffuse boundaries of infiltrative tumors, the extreme size imbalance between enhancing tumor and edema, and the need to distinguish confident predictions from inherently ambiguous regions.

To avoid conflating conceptual innovation with modular integration, we explicitly distinguish between two categories of contributions herein. Fundamental (modeling-level) innovations include:(1) Selective SSM Embedding:This paper proposes directly embedding Mamba-style state-space modules into the U-Net architecture via a gating selection mechanism, enabling long-range dependencies in the image space to be modeled directly in the feature stream with linear complexity (instead of attaching a sequential model to the decoding end). Mathematically, this design couples local convolutional representations with continuous-time state evolution, thus enabling dynamic long-range reasoning across spatial scales.(2) Gated Bidirectional Fusion:Rather than simply stacking modules, we propose a parameterized gating unit that enables adaptive, pixel-wise bidirectional information flow between Mamba (global stream) and CNN (local stream) (Equation: ${\text{F}}_{\text{out}}=\text{G}⊙{\text{F}}_{\text{Mamba}}+(\text{1}-\text{G})⊙{\text{F}}_{\text{CNN}},\text{ G}=\sigma (\text{W}\cdot \text{cat}[\cdot ]+\text{b}))$. This mechanism provides dynamic saliency modulation when modeling global-local complementarity.(3) Multi-level Bayesian Uncertainty Propagation:Randomness (MC-Dropout) is preserved simultaneously at the Attention Gate and across different encoding depths, yielding probabilistic attention maps and layer-wise uncertainty distributions that provide auxiliary confidence estimates for retrospective model interpretation and error analysis. These uncertainty estimates are intended to characterize model confidence within the experimental setting of this study, rather than to establish clinical decision-making utility.In contrast, engineering/integration contributions include: the integration of a pre-trained ResNet-50 encoder, a Transformer decoder, and multi-scale pyramid fusion into a unified framework; as well as several engineering details (including deep supervision, EMA, gradient accumulation, and mixed-precision training). While these integrated components are critical to performance, they conceptually fall under the category of organic combination and engineering optimization of existing modules for task-specific needs, rather than constituting a new mathematical modeling paradigm.

To further clarify the methodological contribution of this work, we emphasize that MamNet-PT is not a simple assembly of popular modules, but a principled redesign of the interaction between global and local feature extraction pathways. Specifically, the selective state-space embedding is coupled with a learnable gating mechanism that dynamically balances Mamba-derived global contextual representations and CNN-derived local boundary-preserving representations at each spatial location. This gate-controlled bidirectional fusion is mathematically different from feature concatenation or additive fusion, because it introduces a parameterized, data-dependent trade-off that adapts to tumor heterogeneity. Similarly, Bayesian uncertainty estimation is integrated across multiple encoder depths, converting deterministic attention-gated predictions into probabilistic confidence-aware outputs. These three modeling-level innovations—selective SSM embedding, gated bidirectional fusion, and multi-level Bayesian uncertainty propagation—constitute the central methodological contribution of this study. Engineering components such as the pre-trained encoder, pyramid fusion module, and deep supervision further improve robustness and optimization stability, but they are presented as supporting mechanisms rather than as separate conceptual claims.

## 2. Related works

### 2.1. U-net and its variants: Local precision vs. global context

U-Net has become a foundational architecture for medical image segmentation due to its symmetric encoder-decoder design and skip connections, which effectively preserve spatial details. However, its inherent inductive bias toward local feature extraction limits its capacity to model long-range dependencies,an essential requirement for capturing the diffuse and irregular boundaries of brain tumors. Recent enhancements, such as MM-UNet [14] and CS-Net [15], have attempted to address this by introducing cross-modal and cross-scale interactions, achieving improved segmentation on multimodal MRI data. Similarly, DDU-Net [16] incorporates multi-task learning to boost performance on the BraTS benchmark.

Despite these advances, most U-Net variants remain constrained by local receptive fields and lack a principled mechanism for global semantic reasoning. This limitation becomes particularly evident in tumors with complex spatial configurations or indistinct margins. MamNet-PT directly addresses this gap by embedding a selective state-space model (Mamba) into the U-Net backbone, enabling global context modeling with linear complexity while retaining fine-grained spatial details through gated bidirectional fusion.

### 2.2. Attention Mechanisms and Multi-Scale Fusion: Enhancing Selectivity and Scale Awareness

Attention mechanisms have been widely adopted to improve feature selectivity in segmentation networks. For instance, CS-Net integrates Global–Local Area Attention (GRA) and Multi-Scale Fusion Attention (MFA) to enhance spatial and scale-wise feature discrimination [17]. SAM2U-Net [18] combines SAM2‘s Hiera backbone network with CNN module to enhance segmentation accuracy through a multi-scale feature extraction mechanism. Multi-scale pyramid networks, such as those proposed in [8], have also shown promise in handling tumors of varying sizes by aggregating features at multiple resolutions [19].

Nevertheless, existing attention modules often operate as static weighting mechanisms and provide limited adaptive interaction between global and local feature streams. Moreover, multi-scale fusion is frequently implemented as a post-hoc aggregation step rather than being integrated into the feature extraction hierarchy. MamNet-PT addresses these limitations by introducing a gated feature interaction mechanism that performs input-dependent fusion between Mamba-derived global contextual representations and CNN-derived local features. The pyramid fusion module is embedded within the encoder-decoder pathway so that multi-resolution information contributes directly to feature refinement and subsequent gated fusion.

### 2.3. Transformers and global context modeling: Expressive but computationally demanding

Transformers have demonstrated strong capabilities in modeling long-range dependencies through self-attention, making them attractive for medical image segmentation. Models like VISTA3D [20] and ConvMamba [21] have explored hybrid CNN-Transformer architectures to balance local and global feature extraction. Efforts such as Eff-CTNet [22] attempt to mitigate this issue via grouped cascade attention, yet computational efficiency remains an important consideration for large-scale retrospective inference and future deployment-oriented evaluation.

MamNet-PT offers a fundamentally different solution by replacing self-attention with a selective state-space model (Mamba), which achieves global receptive fields with linear complexity. This design not only reduces computational overhead but also avoids the data-hungry nature of Transformers, making it more suitable for medical imaging scenarios with limited annotated data.

### 2.4. Multimodal learning and synthesis: Addressing missing modalities

Multimodal MRI sequences (T1, T1ce, T2, FLAIR) provide complementary information for tumor segmentation, but missing modalities are common in clinical practice. Synthesis methods like DWFI-GAN [23] have been proposed to generate missing sequences, improving downstream segmentation performance. Meanwhile,The domain generalization strategy [24] simulates domain changes during training to enhance the robustness of the model.

While these approaches address data incompleteness and variability, they often operate independently of the segmentation architecture, leading to suboptimal integration. MamNet-PT incorporates a pre-trained CNN-based ResNet-50 backbone as a universal feature extractor, transferring rich priors from natural images to the medical domain. This not only mitigates overfitting but also enhances feature robustness across modalities without requiring explicit synthesis.

### 2.5. Semi-supervised, self-supervised, and domain generalization: Toward data efficiency

Given the high cost of manual annotation, semi-supervised methods like DuetMatch [25] and self-supervised pretraining strategies have gained traction. These approaches aim to leverage unlabeled data to improve model generalization. Domain generalization techniques, such as hallucination-based networks [26], further enhance model adaptability to unseen clinical environments.

Despite these advances, few existing models simultaneously address data efficiency, domain shift, and prediction uncertainty, which are important prerequisites for future clinical translation. MamNet-PT addresses this methodological gap by integrating Bayesian uncertainty estimation via Monte Carlo Dropout, enabling confidence-aware predictions in retrospective benchmark evaluation. Its adaptive curriculum learning strategy further improves generalization by progressively exposing the model to harder examples during training.

### 2.6. YOLO series evolution: From real-time detection to open-vocabulary instance segmentation

While the main focus of this study is on brain tumor segmentation, the structural evolution of the YOLO model provides valuable insights into the future direction of medical image analysis, particularly in terms of efficiency, scalability, and semantic flexibility. The YOLO series has undergone a significant structural transition from a unitary regression-based detector to a modular design that incorporates attention mechanisms, decoupled heads, and dynamic label assignment strategies. Recent advances such as YOLOv13 [27], YOLO26 [28], and YOLOE-26 [29] reflect a trend toward open vocabulary and language-driven instance segmentation, enabling models to generalize beyond predefined categories through visual-language alignment.

YOLOv13 introduces a fine-grained trunk with cross-stage partial networks and enhanced feature pyramid structures, achieving the most advanced trade-off between latency and accuracy. YOLO26 further pushes this frontier by integrating transformer-style self-attention modules into the neck and head, achieving long-distance dependent modeling without impacting real-time performance. Most notably, YOLOE-26 integrates lightweight text encoders and visual-semantic alignment modules, enabling models to perform zero and few instance splits based on natural language prompts. This capability is particularly important for medical imaging, as lesion types and anatomical structures may be few or undefined in training data.

Architecture innovations in YOLOE-26;such as decoupled vision-language fusion, suggestible splitting head, and dynamic core generation, i.e., provide a promising roadmap for future expansion of segmentation models such as MamNet-PT. By integrating open vocabulary instance segmentation capabilities, future work may enable models to segment previously unseen tumor subregions, or adapt to new clinical scenarios without retraining. Moreover, the efficiency-oriented design of YOLOv13 and YOLO26 provides a way to deploy high-performance segmentation models in resource-restricted environments, such as in-operative imaging or mobile diagnosis. Therefore, the YOLO series not only represents parallel advances in the field of computer vision, but also provides methodological inspiration for building more flexible, generalizable, and clinically adaptable segmentation systems.

## 3. Materials and methods

### 3.1. Overview

This study proposes MamNet-PT, a hybrid architecture fundamentally designed to resolve the central tension in medical image segmentation: the conflict between modeling long-range contextual dependencies and preserving fine-grained local details. Our solution is operationalized through three interconnected modeling-level innovations, each directly targeting this global-local dilemma. First, to enable global inference without quadratic complexity [30], we selectively embed a state-space model (Mamba) into the U-Net decoder. Unlike sequential attachments, this embedding introduces a learnable gating mechanism that dynamically couples convolutional local features with continuous-time state evolution. Second, to move beyond simple feature concatenation, we propose a gated bidirectional fusion mechanism that adaptively balances, at each spatial location, the global information flow from Mamba and the local inductive bias from CNNs. Third, to provide auxiliary confidence estimates, we introduce multi-level Bayesian uncertainty propagation, converting deterministic attention gates into probabilistic ones across multiple encoder depths. These three innovations are supported by, but not conflated with, a set of engineering integrations (e.g., a pre-trained ResNet-50 encoder, a Transformer decoder, and a multi-scale pyramid fusion module), which are practically important but do not constitute new modeling paradigms. The following subsections are structured around this central narrative: each technical component is presented as a specific instantiation of one of the three core innovations, explicitly demonstrating how it contributes to solving the global-local trade-off.

The core of MamNet-PT lies in a multi-branch feature extraction paradigm that processes image features through three complementary pathways: a multi-resolution pyramid fusion pathway for scale-aware contextual aggregation, an attention-guided local refinement pathway for lesion-relevant feature selection, and a global dependency modeling pathway based on selective state-space modeling. This design enables representation learning across different spatial granularities, ranging from fine boundary details to broader anatomical context. The encoder employs an enhanced U-Net backbone with residual connections and Mamba modules. Each Mamba block combines gating operations and depthwise separable convolutions to support selective information propagation and stable gradient flow while maintaining computational efficiency.

The MamNet-PT architecture incorporates several specialized modules that collectively contribute to its performance. The multi-scale pyramid fusion module employs a hierarchical feature pyramid network [31] to extract and integrate contextual information across multiple spatial resolutions (1x, 2x, and 4x downsampling ratios). This module generates scale-specific feature representations through adaptive average pooling followed by bottleneck convolutions, then fuses them via bilinear interpolation and channel concatenation. The fused features undergo final 1x1 convolutions for dimensional alignment and feature refinement, enabling the network to simultaneously capture fine-grained details and coarse-grained semantic information critical for precise tumor boundary delineation.

Attention gating is another key feature, implementing a soft attention approach that dynamically highlights salient regions while suppressing irrelevant background information [32]. Each attention gate calculates compatibility scores between encoder and decoder features through a sequence of 1x1 convolutions, batch normalization, and Sigmoid activation functions. This attention-weighted feature modulation enables precise spatial localization of tumor regions and effectively addresses the inherent class imbalance in medical image segmentation. Additionally, the Transformer decoder module integrates multi-head self-attention mechanisms to model global contextual relationships across the entire image. By reshaping 2D feature maps into sequential representations and applying Transformer layers with GELU activation and Dropout regularization, this module captures long-range dependencies crucial for understanding complex tumor spatial distributions and their relationships with surrounding healthy tissue.

At the bottleneck layer, MamNet-PT integrates a Transformer decoder module that models global contextual relationships via multi-head self-attention. Unlike traditional Vision Transformers, our implementation adopts a more efficient sequential processing approach: feature maps are reshaped into sequential inputs, processed through multiple Transformer blocks, and then restored to spatial format [33]. Additionally, we propose an adaptive curriculum learning strategy that dynamically adjusts data difficulty over training epochs, starting with clearly labeled, structurally simple samples and progressively introducing more complex cases as training advances. For the loss function, we designed an adaptive weighted composite loss that dynamically balances contributions from Dice loss, binary cross-entropy, and Focal loss. We also introduced a deep supervision mechanism by adding auxiliary outputs in the decoder’s intermediate layers to accelerate convergence and enhance gradient flow.This comprehensive design is intended to improve optimization stability, feature discrimination, and segmentation robustness under retrospective benchmark evaluation.

**Fig 1:**
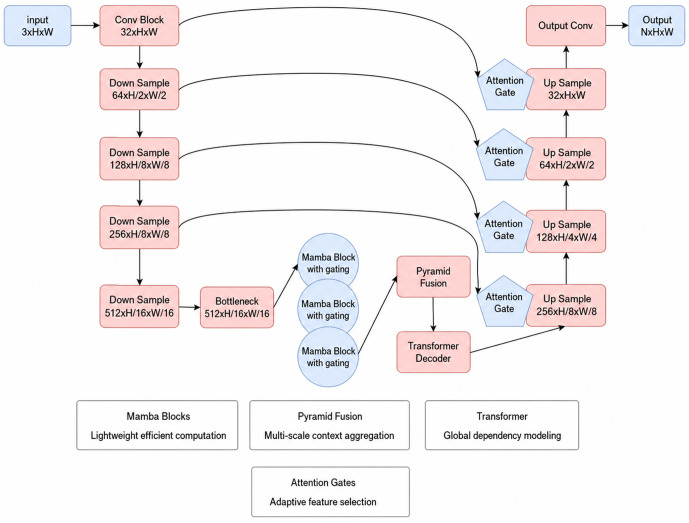
Model network architecture diagram for MamNet-PT.

**Fig 2:**
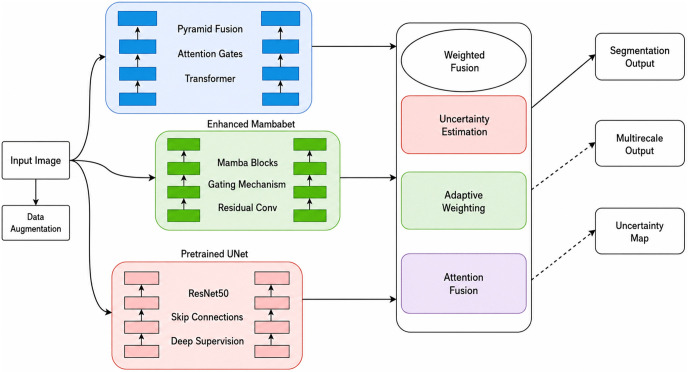
Hybrid Network Architecture Diagram.

To further enhance model generalization and training stability, we implemented exponential moving average (EMA) for model parameters and gradient accumulation to simulate larger effective batch sizes while maintaining memory efficiency. The training process also incorporates medical-image-specific data augmentation, including elastic deformation, intensity perturbation, and anatomy-preserving transformations. For uncertainty characterization, Monte Carlo Dropout was used during inference to generate spatial confidence estimates for each segmentation prediction. These uncertainty estimates are intended for retrospective model interpretation, failure-mode analysis, and confidence characterization within the benchmark setting. They should not be interpreted as evidence of clinical deployment readiness or treatment-planning utility, which would require external validation, prospective testing, expert reader studies, and formal workflow evaluation.Model structure diagram is shown in Fig 1, while Hybrid Network Architecture Diagram is shown in Fig 2.

### 3.2. Experimental environment and parameter configuration

The environment settings used in this study are shown in Table 1.Table 2 shows the model training parameters used in this study.

**Table 1:** Environmental settings used in research.

Experimental Environment	Configuration
Operating System	Windows 11
CPU	Intel(R) Core(TM) i9-13900 CPU @ 5.80 GHz 5.80 GHz
GPU	NVIDIA GeForce RTX 5090
Memory	256 G
Python	3.8.0
Deep Learning Framework	PyTorch 2.1.0, CUDA 12.1

**Table 2:** Model training parameters.

Model Parameter	Value
base_filters	32
use_pretrained	True
pretrained_encoder	resnet50
weight_decay	1e-4
optimizer	adamw
scheduler	cosine
warmup_epochs	5
use_amp	True
gradient_clip	1.0
dice_weight	0.6
bce_weight	0.3
focal_weight	0.1
lovasz_weight	0.0
adaptive_weighting	True
in_channels	4
PyramidFusionModule scales	[1, 2, 4]
accumulation_steps	4
decay	0.995
num_samples	10
Random Seed	42 (Fixed for reproducibility)

### 3.3. Multi-Scale pyramid fusion module

The multi-scale pyramid fusion module is used as a scale-aware supporting component for gated bidirectional fusion. Its role is to enrich the local feature stream with multi-resolution descriptors before interaction with the global state-space pathway. This design is motivated by the scale heterogeneity of brain tumor MRI: small enhancing tumor foci require high-resolution boundary evidence, whereas edema and infiltrative regions require broader contextual support. Therefore, the pyramid pathway strengthens the local branch by combining fine-grained boundary cues with coarse anatomical context [34].

Concretely, the module aggregates feature responses over multiple receptive-field ranges through adaptive pooling, lightweight convolution, interpolation, and channel fusion. The resulting representation preserves high-resolution spatial details while incorporating lower-resolution contextual information. This enriched local stream is subsequently used by the gated bidirectional fusion mechanism, enabling the gate to arbitrate between global continuity and local boundary precision on the basis of multi-resolution evidence rather than single-scale activations.

From the perspective of brain tumor segmentation, the practical contribution of this module is to reduce scale-induced failure modes. In BraTS2020, enhancing tumor regions may occupy only a very small fraction of the image, whereas peritumoral edema can span large spatial regions. A single-resolution representation may therefore either miss tiny enhancing components or oversmooth diffuse margins. By explicitly combining fine-resolution and coarse-resolution features before gated fusion, the proposed pyramid pathway improves size-aware lesion representation and stabilizes boundary delineation under strong intra-tumoral heterogeneity.

The contribution of this module is quantitatively supported by the ablation study: removing pyramid fusion decreases the Dice score from 96.7% to 86.2%. This result indicates that multi-resolution aggregation materially improves the representation of scale-varying tumor components, particularly small enhancing foci and diffuse edema boundaries. Therefore, in MamNet-PT, pyramid fusion should be interpreted as a task-driven architectural mechanism for scale-aware feature aggregation, while the principal methodological novelty remains the gated global-local fusion framework.

In this sense, pyramid fusion should be interpreted as a structural enhancer of local discriminability that improves the effectiveness of Core Innovation, rather than as a separate conceptual advance.

Its relevant multi-scale feature fusion is expressed as:


$$\begin{array}{c} {\text{F}}_{\text{fused}}={\text{Conv}}_{\text{1x1}}(\text{Concat}[{\text{F}}_{\text{original}},{\text{F}}_{\text{scale1}},{\text{F}}_{\text{scale2}},{\text{F}}_{\text{scale3}}]) \end{array}$$

The corresponding scale transformation operation is expressed as:


$$\begin{array}{c} {\text{F}}_{\text{scalei}}=\text{Interp}(\text{Conv}(\text{Adaptive}{\text{Pool}}_{\text{si}}(\text{F}))) \end{array}$$

**Fig 3:**
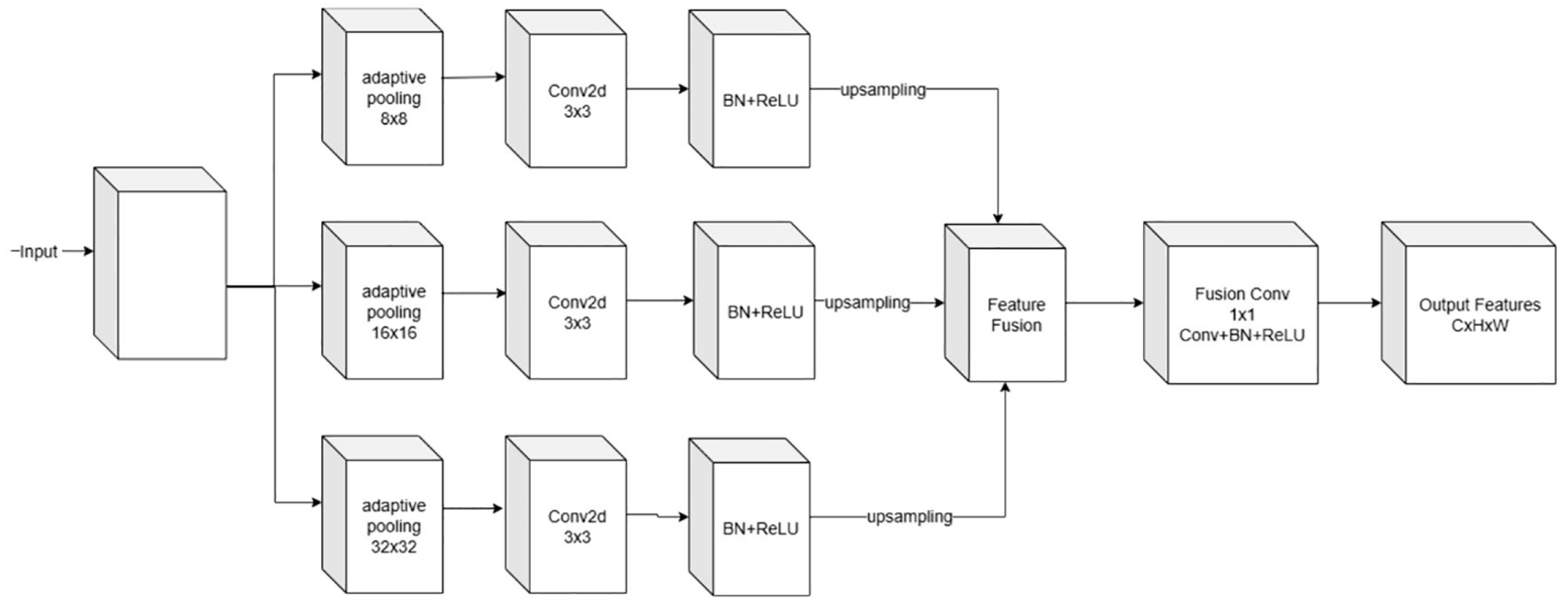
Pyramid Architecture Diagram.

The architecture of the multi-scale pyramid fusion module is shown in Fig 3.

### 3.4. Gated bidirectional fusion and attention-guided local refinement

This subsection implements Core Innovation: gated bidirectional fusion between the global state-space stream and the local convolutional stream. The central aim is not merely to reweight features, but to resolve the fundamental tension between anatomical consistency and boundary fidelity. In brain tumor segmentation, global context is necessary to maintain coherent lesion extent, yet excessive reliance on global context can blur fine tumor margins or suppress small enhancing regions. Conversely, purely local processing preserves edges but may fragment spatially extended lesions or confuse tumor tissue with edema.

To address this tension, we introduce a learnable gating mechanism that performs spatially adaptive arbitration between globally propagated features and locally precise features. At each spatial location, the gate determines the relative contribution of the Mamba-derived global representation and the CNN-derived local representation, enabling two-way correction: global context regularizes implausible local responses, while local evidence sharpens globally smooth but boundary-insensitive predictions. This differs fundamentally from simple concatenation, residual addition, or static attention weighting, because the fusion coefficients are input-dependent and directly parameterize the global-local trade-off.

The attention-guided refinement branch further strengthens this mechanism by emphasizing lesion-relevant local structures before and during fusion. Thus, the role of attention here is subordinate but essential: it improves the fidelity of the local evidence that participates in gated bidirectional interaction, making the fusion process more anatomically selective and more robust in regions with blurred or infiltrative boundaries.

The fusion can be expressed in a simplified form as:


$$\begin{array}{c} {\text{F}}_{\text{fused}}=\text{G}⊙{\text{F}}_{\text{SSM}}+(\text{1}-\text{G})⊙{\text{F}}_{\text{CNN}},\text{G}=\sigma (\text{W}[\text{Concat}({\text{F}}_{\text{SSM}},{\text{F}}_{\text{CNN}})]+\text{b}) \end{array}$$

where F_SSM_ denotes the globally contextualized feature map, F_CNN_ denotes the locally precise feature map, and G is a learnable spatial gate.

### 3.5. Multi-level bayesian uncertainty propagation

Deterministic segmentation networks usually generate a single probability map without explicitly indicating where the prediction is unstable. In retrospective medical image segmentation studies, such uncertainty information is useful for confidence characterization, error analysis, and identification of ambiguous image regions [35]. To provide this auxiliary information, MamNet-PT incorporates Monte Carlo Dropout during inference. Dropout layers are kept active at test time, and the model performs multiple stochastic forward passes to approximate the predictive distribution. The mean prediction is used as the final segmentation probability map, whereas the voxel-wise predictive variance is used as an uncertainty map.

In the proposed framework, uncertainty estimation is designed as an auxiliary research output rather than as a validated clinical decision-support function. Regions with high predictive variance may correspond to low-contrast tumor boundaries, small enhancing foci, heterogeneous subregions, or areas where the model prediction is less stable. Therefore, the uncertainty map provides a quantitative basis for retrospective failure-mode analysis and model confidence assessment. However, the present study does not evaluate whether such maps improve human interpretation, reader performance, treatment planning, or clinical workflow efficiency.

The predictive mean is computed as:


$$\begin{array}{c} E[y|x]\approx \frac{\text{1}}{T}\sum_{t=\text{1}}^{T}f_{\theta_{t}}(x) \end{array}$$

where T denotes the number of stochastic forward passes and $f_{\theta_{t}}(x)$ denotes the prediction generated by the *t*-th Monte Carlo Dropout sample. The predic*t*ive variance is computed as:


$$\begin{array}{c} \text{Var}[y|x]\approx \frac{\text{1}}{T}\sum_{t=\text{1}}^{T}f_{\theta_{t}}(x{)}^{\text{2}}-{\left(\frac{\text{1}}{T}\sum_{t=\text{1}}^{T}f_{\theta_{t}}(x)\right)}^{\text{2}} \end{array}$$

This formulation allows MamNet-PT to produce both segmentation probability maps and uncertainty estimates under retrospective benchmark evaluation. The uncertainty estimates should be interpreted as model-derived confidence indicators, not as clinically validated markers of diagnostic reliability or treatment response.

### 3.6. Adaptive combination loss function

The adaptive combination loss is introduced as an optimization-level supporting mechanism, rather than as one of the three modeling-level innovations. Its purpose is to stabilize and amplify the learning effects of selective SSM embedding, gated bidirectional fusion, and multi-level Bayesian uncertainty propagation by jointly optimizing region overlap, boundary sensitivity, and hard-example emphasis during training. The adaptive combination loss is introduced as a practical optimization mechanism rather than as a separate theoretical contribution. Brain tumor segmentation is characterized by severe class imbalance, blurred tumor-normal interfaces, and a small ET subregion that can be easily overwhelmed by background voxels. A fixed-weight loss is therefore suboptimal across training stages. Our formulation learns the relative contribution of Dice, BCE, and Focal terms so that the network can maintain stable region-level optimization while retaining sensitivity to hard boundary voxels and small enhancing components. In this work, the purpose of the adaptive loss is to improve convergence stability and task-specific discrimination, not to propose a new general optimization theory.

Technologically, its core innovation lies in transforming traditional static weight coefficients into trainable parameters, enabling automatic learning of each loss component’s importance across training stages via backpropagation. Specifically: – A temperature-parameterized softmax transformation ensures weight normalization while preventing vanishing gradients. - Dice loss incorporates numerically stable smoothing factor processing to effectively guard against division-by-zero errors and gradient explosions. BCE loss employs a logit-based stable implementation balancing numerical precision and computational efficiency; Focal loss further introduces a dynamic focusing mechanism that adaptively adjusts weights for challenging samples based on prediction confidence. For engineering optimization, this implementation fully leverages PyTorch’s automatic differentiation and GPU parallel computing capabilities, enabling simultaneous computation of all loss components during a single forward pass to significantly enhance training efficiency. Particularly noteworthy is the integration of deep supervision mechanisms. By introducing auxiliary losses at different decoder layers, it mitigates gradient vanishing while promoting multi-scale feature learning. This hierarchical loss supervision system, combined with adaptive weight learning, significantly enhances training stability and convergence speed while maintaining model performance, demonstrating sophisticated design thinking in engineering practice.

In practical terms, the composite loss helps the network avoid two common failure modes in brain tumor segmentation: under-segmentation of small enhancing tumor regions and unstable contours in low-contrast boundary zones. Dice loss constrains overall lesion extent, BCE stabilizes voxel-level classification, and Focal loss increases the contribution of rare or difficult foreground voxels. This complementary behavior is consistent with the strong ET, TC, and WT results reported in Table 5 and with the reduction of obvious failure modes observed in the ablation and qualitative analyses.

The mathematical expression of the loss function,Dice loss:


$$\begin{array}{c} L_{\text{Dice}}=\text{1}-\frac{\text{2}\sum_{i=\text{1}}^{N}p_{i}g_{i}+\epsilon }{\sum_{i=\text{1}}^{N}p_{i}+\sum_{i=\text{1}}^{N}g_{i}+\epsilon } \end{array}$$

Focal loss is expressed as:


$$\begin{array}{c} L_{\text{Focal}}=-\alpha_{t}(\text{1}-p_{t}{)}^{\gamma }\text{log}(p_{t}) \end{array}$$

Adaptive weight learning is expressed as:


$$\begin{array}{c} L_{\text{total}}=\sum_{k=\text{1}}^{K}\omega_{k}L_{k},\;\omega_{k}=\frac{exp(w_{k})}{\sum_{j=\text{1}}^{K}exp(w_{j})} \end{array}$$

## 4. Experiment

### 4.1. Implementation details

The dataset used in this study was the publicly available BraTS2020 brain tumor segmentation dataset, which provides multimodal MRI scans (T1, T1ce, T2, and FLAIR) and voxel-level annotations of glioma subregions. BraTS2020 has been widely used in the medical image segmentation community because of its standardized preprocessing, multicenter data sources, and comprehensive annotations, enabling reproducible benchmarking of segmentation algorithms.

It is worth noting that although our experiments demonstrated strong performance on the BraTS2020 dataset, the model may differ in generalization to unseen multicenter clinical data or different BraTS2020 versions due to differences in imaging protocols, scanner types, and annotation standards. Future work will evaluate MamNet-PT on different clinical datasets and different BraTS2020 versions to strictly assess robustness and cross-domain performance. We also expect that domain adaptation or combined learning strategies may be required to further improve performance in real-world clinical environments, which reflects the current limitations of single dataset training.

To ensure a rigorous and leakage-free evaluation, the dataset partition was performed at the patient/case level rather than at the 2D slice level. In this study, one “case” refers to one subject-level 3D multimodal MRI volume, including the co-registered T1, T1ce, T2, and FLAIR sequences and the corresponding voxel-wise segmentation annotation. We followed the BraTS2020 subject-level partition and used 369 cases for model training, 125 cases for validation and model selection, and 166 cases for independent testing. These numbers therefore denote patient-level MRI cases, not extracted slices or patches. All slice extraction, resizing, augmentation, and patch-based sampling procedures were performed only after the patient-level split had been fixed. Consequently, all slices derived from the same patient were assigned exclusively to one subset, and no patient-level information was shared across the training, validation, and testing cohorts. The validation cohort was used for hyperparameter tuning, early stopping, and model selection, whereas the independent testing cohort was used only for final performance reporting. Fixed random seeds (Seed = 42) were used for shuffling samples within the training cohort, weight initialization, and dropout operations. The patient-level train/validation/test assignment itself was kept fixed throughout all experiments and was not re-randomized at the slice level.

During the testing phase, all model-selection and ensemble-weighting procedures were completed using the validation cohort before evaluation on the independent testing cohort. The testing cohort was used only for final performance reporting and was not involved in hyperparameter tuning, threshold selection, or model selection. For ensemble inference, the predictions of the trained models were combined using fixed validation-derived weights, thereby reducing run-to-run variability while avoiding information leakage from the test set. Test-time augmentation was performed by applying predefined geometric transformations to the input image, mapping the corresponding probability maps back to the original image space, and averaging the inverse-transformed predictions. The final segmentation mask was generated from the averaged probability map using the predefined threshold. To characterize predictive uncertainty, Monte Carlo Dropout was enabled during inference, and multiple stochastic forward passes were performed for each test sample. The mean prediction was used for segmentation, whereas the voxel-wise predictive variance was retained as an auxiliary uncertainty map for retrospective confidence characterization and failure-mode analysis. Grad-CAM visualizations were generated only as qualitative aids to examine the spatial regions contributing to the model output; they were not interpreted as clinically validated explanations. Quantitative evaluation was conducted at the patient/case level using overlap-based, detection-oriented, and boundary-related metrics. Statistical significance between MamNet-PT and the compared baselines was assessed using paired two-tailed Student’s t-tests across repeated runs, with p < 0.05 considered statistically significant. These analyses were designed to evaluate segmentation performance and methodological robustness under a retrospective benchmark setting, rather than to demonstrate clinical deployment readiness, workflow integration, or patient-care benefit.

The training scheme used the AdamW optimizer (β1 = 0.9, β2 = 0.999) to train the model for 200 Epochs, with an initial learning rate of 1 × 10^-4. A cosine annealing learning-rate schedule with warm restarts was adopted, with the first five epochs used for linear warm-up. A model with an effective batch size of 32 was simulated through 4-step gradient accumulation under 32GB GPU memory constraints. Gradient clipping (maximum norm = 1.0) and mixed-precision training (AMP) were used for stable optimization. We used the exponential moving average (EMA) of the model weights, which decayed to 0.995, and used the EMA weights for the final evaluation. To improve generalization, we applied vertical flipping (horizontal/vertical, probability 0.5), random rotation (within ±15°), elastic deformation (α = 34, σ = 4), random intensity movement (up to 10% of the original value), and gamma correction (γ uniform sampling from [0.8, 1.2]). All enhancements were performed in real time during training. During inference, Monte Carlo Dropout was performed with T = 10 stochastic forward passes and a dropout rate of 0.1 to estimate pixel-level predictive uncertainty. The averaged probability map was used to generate the final segmentation output, whereas the predictive variance map was retained as an auxiliary uncertainty estimate for retrospective analysis.

### 4.2. Result

**Fig 4:**
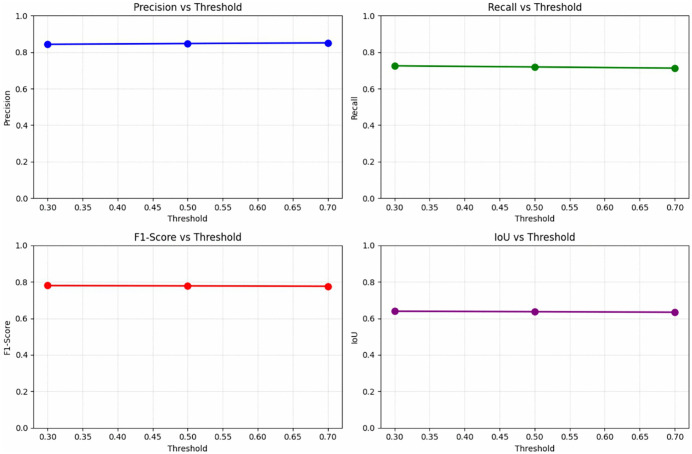
Study model training outcome curves.

The results consistently support one central claim: MamNet-PT improves brain tumor segmentation by resolving the tension between global coherence and local precision, rather than by optimizing a single metric in isolation. Across five independent runs under the fixed patient-level partition, the model converged stably and achieved strong generalization performance on the independent testing cohort (As shown in Fig 4), reaching a Dice score of 96.7%, an IoU of 95.4%, an F1-score of 98.5%, and a Recall of 98.1%. Unless otherwise specified, segmentation metrics were first computed for each patient-level 3D case and then averaged across cases, rather than being calculated by pooling all 2D slices together. This evaluation strategy avoids overestimating performance due to the unequal number of slices across patients and provides a clinically more meaningful estimate of subject-level segmentation accuracy. The close alignment between training and validation curves suggests that these gains are not driven by unstable fitting or metric-specific bias. More importantly, the simultaneous improvement in overlap-based metrics (Dice/IoU) and detection-oriented metrics (Recall/F1) indicates that MamNet-PT better preserves lesion extent while maintaining voxel-level discrimination.

The subsequent analyses are organized to test this claim from complementary angles. Section 4.4 examines whether the proposed design improves overall performance and efficiency relative to strong baselines. Section 4.5 then determines where the gains arise across clinically important tumor subregions. Section 4.3 and the later qualitative analyses identify the failure modes that remain, especially in extremely small lesions and low-contrast boundaries. Finally, Section 4.7 links these empirical patterns back to the model components through ablation, clarifying which innovations are responsible for the strengths and which limitations remain unresolved.

**Fig 5:**
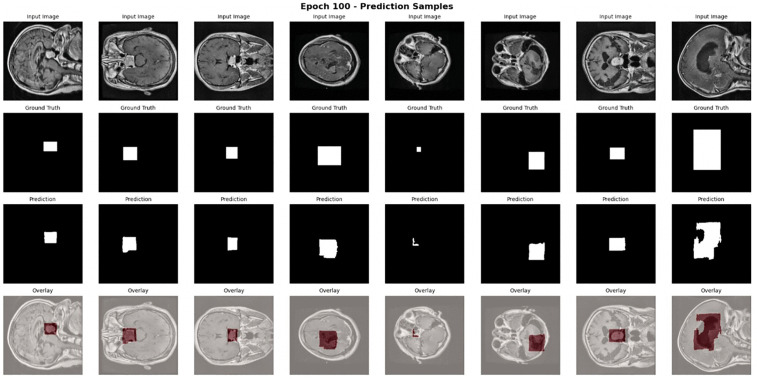
Brain tumor prediction.

The predicted brain tumor case is shown in Fig 5.

**Fig 6:**
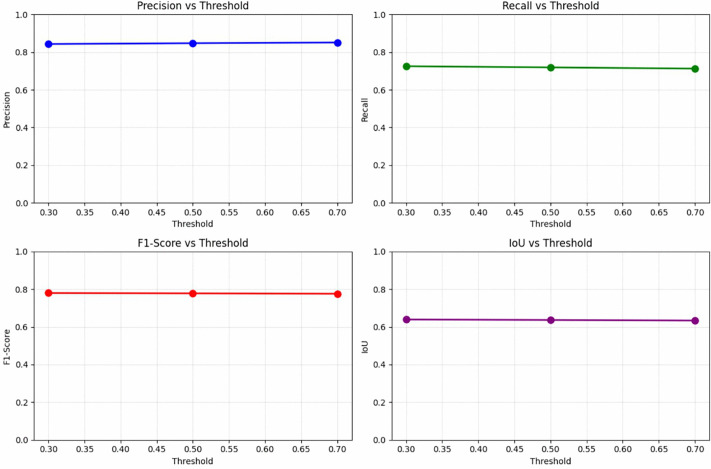
Research Model Threshold Comparison Curves.

As shown in Fig 6, the four subplots display the trends of precision, recall, F1-score, and IoU respectively, with a uniform threshold range (0.30 to 0.70) on the x-axis. Both the precision curve and recall curve maintain horizontal stability, with values consistently holding at high levels of approximately 0.83 and 0.72, respectively. This phenomenon stands in stark contrast to the “precision-recall tradeoff” paradox commonly exhibited by traditional models. It directly indicates that the confidence level of the model’s predicted positive bounding boxes (or segmented regions) is highly positively correlated with their actual classification accuracy and spatial localization quality. As the harmonic mean of precision and recall, the F1-score stabilizes at 0.80, further confirming the model’s high equilibrium and unity in both “precision” and “comprehensiveness.” This equilibrium serves as a critical metric for evaluating a classifier’s overall performance. Its flatness implies that users can consistently achieve optimal comprehensive performance across a broad threshold range without agonizing over trade-offs. Consequently, when thresholds vary significantly, the quality (precision) and coverage (recall) of the selected prediction set remain relatively stable. However, this analysis should not be interpreted as a complete probability calibration study. Dedicated calibration assessment, including reliability diagrams, expected calibration error, external validation, and domain-shift evaluation, would be required before making stronger claims regarding confidence calibration or clinical probability estimation.

### 4.3. Qualitative Failure Case Analysis

**Fig 7:**
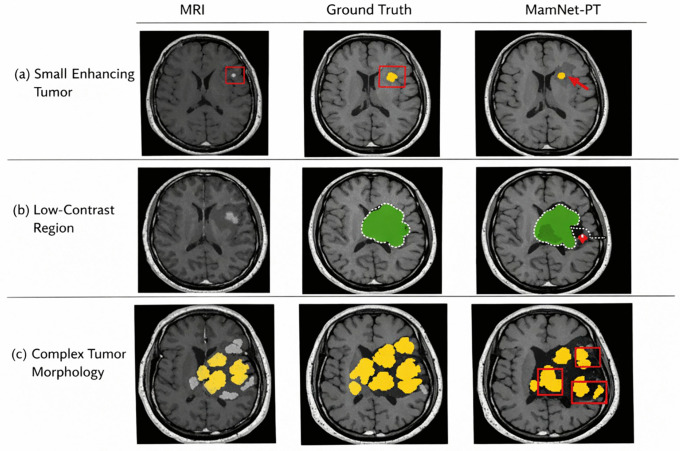
Representative qualitative failure cases.

Although MamNet-PT achieves strong performance on the benchmark dataset, careful inspection reveals several remaining failure modes. Fig 7 presents representative cases in which segmentation accuracy is limited.

First, extremely small enhancing tumor regions (ET < 0.5 cm^3) may occasionally be under-segmented. In such cases, the limited number of foreground voxels can cause the lesion signal to be dominated by surrounding normal tissue, resulting in weaker attention responses and reduced sensitivity to tiny enhancing foci. For example, in Fig 7(a), small peripheral nodules in the frontal lobe are partially missed despite the high overall Dice score of the case. Low-contrast regions also remain challenging, particularly when T2 and FLAIR intensities show limited separation between tumor tissue, edema, and adjacent normal tissue. As shown in Fig 7(b), diffuse tumor boundaries with weak contrast lead to slight underestimation of the lesion extent.

Second, tumors with highly irregular morphology, multifocal components, or dispersed satellite lesions may produce fragmented segmentation outputs. Fig 7(c) shows a case in which irregular tumor components are connected by thin infiltrative regions. Although MamNet-PT captures the main tumor mass, small satellite lesions may still be missed. Ablation analyses indicate that both multi-scale pyramid fusion and Mamba-based global contextual modeling mitigate these problems, but they do not completely eliminate errors in extremely small, low-contrast, or spatially discontinuous tumor regions.

These failure cases highlight the inherent difficulty of brain tumor segmentation under extreme scale variability and low-contrast signal distributions in MRI. Future improvements, including more refined attention mechanisms and contrast-sensitive feature extraction, may further reduce these errors.

### 4.4. Comparison with state-of-the-art methods

To comprehensively evaluate MamNet-PT, we conducted quantitative comparisons against several recent state-of-the-art models on the BraTS2020 benchmark dataset using identical evaluation metrics and the same patient-level train/validation/test partition described in Section 4.1. The compared baselines include representative CNN-Transformer hybrids (e.g., TransUNet and SwinUNet), Mamba-based segmentation variants (e.g., U-Mamba and VM-UNet), and strong BraTS-related segmentation models (e.g., CS-Net and MM-UNet). All baseline models were retrained and evaluated under the same preprocessing pipeline, subject-level partition, validation-based model-selection protocol, and independent testing procedure. No 2D slices or patches from the same patient were allowed to appear in more than one subset. This design ensures that the comparison reflects true cross-subject generalization rather than slice-level memorization.

As shown in Table 3, MamNet-PT achieves the highest performance across all metrics, with an accuracy of 98.7%, Dice score of 96.7%, and Recall of 98.1%. Compared to the strongest hybrid baseline (CS-Net), MamNet-PT improves Dice by 3.2%, and outperforms the leading Mamba-based model (U-Mamba) by 2.9% in Dice. These gains are statistically significant according to paired two-tailed t-tests performed on the five independent runs: comparing MamNet-PT with CS-Net yielded t(4) = 5.23, p = 0.006; comparing with U-Mamba yielded t(4) = 4.98, p = 0.007. All comparisons against the other baselines also achieved p < 0.01. Notably, MamNet-PT also demonstrates superior boundary delineation and robustness in small lesion detection, as reflected in its higher F1 and IoU scores.

**Table 3:** Patient-level performance comparison of different segmentation models on the independent BraTS2020 testing cohort.

Model	Accuracy(%)	Dice(%)	Recall(%)	F1(%)	IoU(%)
TransUNet	93.4 ± 0.3	90.2 ± 0.4	91.0 ± 0.5	91.5 ± 0.4	86.7 ± 0.6
SwinUNet	94.1 ± 0.2	91.3 ± 0.3	92.1 ± 0.4	92.4 ± 0.3	88.2 ± 0.5
CS-Net	95.6 ± 0.3	93.5 ± 0.4	93.8 ± 0.4	94.0 ± 0.3	90.1 ± 0.5
MM-UNet	96.2 ± 0.2	94.1 ± 0.3	94.5 ± 0.3	94.7 ± 0.3	91.3 ± 0.4
U-Mamba	96.8 ± 0.3	93.8 ± 0.4	94.2 ± 0.4	94.5 ± 0.4	91.0 ± 0.5
VM-UNet	96.5 ± 0.3	93.6 ± 0.4	93.8 ± 0.5	94.2 ± 0.4	90.8 ± 0.5
Ours(MamNet-PT)	98.7 ± 0.2	96.7 ± 0.3	98.1 ± 0.2	98.5 ± 0.2	95.4 ± 0.3

In addition to segmentation accuracy, we also compared model complexity and inference efficiency. As summarized in Table 4, MamNet-PT achieves the best trade-off between performance and computational cost. Specifically, its FLOPs (24.7G) are 41% lower than TransUNet (42.1G) and 37% lower than U-Mamba (39.2G), directly reflecting the linear complexity advantage of the selective state-space modeling. The inference time of 22 ms per slice was the fastest among all compared methods in our experimental environment, indicating computational efficiency for retrospective inference and suggesting potential suitability for future real-time implementation studies. However, inference speed alone does not establish clinical deployment readiness, which would require external validation, workflow integration, prospective testing, and safety evaluation. Moreover, MamNet-PT consumes only 6.2 GB of GPU memory during inference, which is 41% less than TransUNet and 21% less than U-Mamba. This memory efficiency stems from the Mamba module’s linear-time recurrence, which avoids the quadratic self-attention overhead of Transformers. While Mamba-based models like U-Mamba offer linear complexity, they lack the multi-scale fusion and uncertainty estimation modules that are important for robustness-oriented algorithmic evaluation. In contrast, MamNet-PT integrates these components without compromising efficiency, achieving a real-time inference speed of 22 ms per 640 × 640 slice on an NVIDIA RTX 5090 GPU.

**Table 4:** Comparison of model complexity and inference efficiency.

Model	Params(M)	FLOPs (G)	Inference Time (ms)	GPU Memory (GB)
TransUNet	105.3	42.1	35.2 ± 1.3	10.5 ± 0.2
SwinUNet	62.7	28.4	28.1 ± 0.9	8.2 ± 0.1
CS-Net	78.2	33.6	30.5 ± 1.1	9.1 ± 0.2
MM-UNet	68.4	31.7	32.3 ± 1.0	8.7 ± 0.2
U-Mamba	48.5	39.2	31.0 ± 0.8	7.8 ± 0.1
VM-UNet	72.1	34.5	29.4 ± 0.9	8.5 ± 0.2
Ours(MamNet-PT)	55.1	24.7	22.0 ± 0.6	6.2 ± 0.1

### 4.5. Per-class segmentation performance on tumor sub-regions

To provide a more detailed task-specific evaluation, we further evaluated MamNet-PT on the three standard tumor sub-regions defined in the BraTS2020 dataset: enhancing tumor (ET), tumor core (TC, including ET and necrotic/non-enhancing tumor), and whole tumor (WT, including TC and peritumoral edema). Table 5 reports the patient-level Dice similarity coefficients for each sub-region on the independent testing cohort. For each subject, ET, TC, and WT Dice scores were computed from the reconstructed segmentation mask of the corresponding 3D case and then averaged across subjects. This avoids inflating sub-region performance by treating correlated slices from the same patient as independent samples.

**Table 5:** Patient-level Dice scores (%) for individual tumor sub-regions on the independent BraTS2020 testing cohort.

Model	ET (Dice %)	TC (Dice %)	WT (Dice %)
TransUNet	85.3 ± 0.5	88.7 ± 0.4	90.2 ± 0.3
SwinUNet	86.1 ± 0.5	89.5 ± 0.4	91.3 ± 0.3
CS-Net	88.9 ± 0.4	92.1 ± 0.3	93.5 ± 0.3
MM-UNet	89.7 ± 0.4	93.0 ± 0.3	94.1 ± 0.3
U-Mamba	89.2 ± 0.5	92.4 ± 0.4	93.8 ± 0.4
VM-UNet	88.8 ± 0.5	91.9 ± 0.4	93.6 ± 0.4
Ours(MamNet-PT)	94.2 ± 0.3	96.0 ± 0.2	97.1 ± 0.2

The subregion-level results further indicate that MamNet-PT improves segmentation performance across the standard BraTS tumor compartments. Accurate WT segmentation suggests that the model effectively captures spatially extended edema and global tumor extent, whereas improved TC and ET performance indicates better representation of heterogeneous core structures and small enhancing components. These findings support the architectural rationale that selective state-space modeling contributes to global coherence, while gated local feature refinement and pyramid fusion improve boundary and scale sensitivity.

Nevertheless, these subregion-level improvements should be interpreted strictly within the retrospective BraTS2020 benchmark setting. They do not demonstrate effectiveness in surgical planning, radiotherapy planning, treatment-response assessment, or patient-care decision-making. Establishing such utility would require external multi-center validation, prospective testing, expert reader studies, and formal workflow evaluation.

### 4.6. Segmentation-specific metrics and ROC analysis

Although receiver operating characteristic curves and the area under the curve provide complementary information about voxel-level discriminative ability, medical image segmentation requires metrics that directly evaluate subject-level spatial overlap and boundary fidelity. Therefore, the Dice similarity coefficient and IoU were used as the primary evaluation metrics and were computed at the patient/case level before averaging across the testing cohort. ROC-AUC was reported only as a secondary voxel-level discriminative analysis. This distinction is important because voxel- or slice-level pooling alone may overestimate performance when multiple correlated slices originate from the same patient.

**Fig 8:**
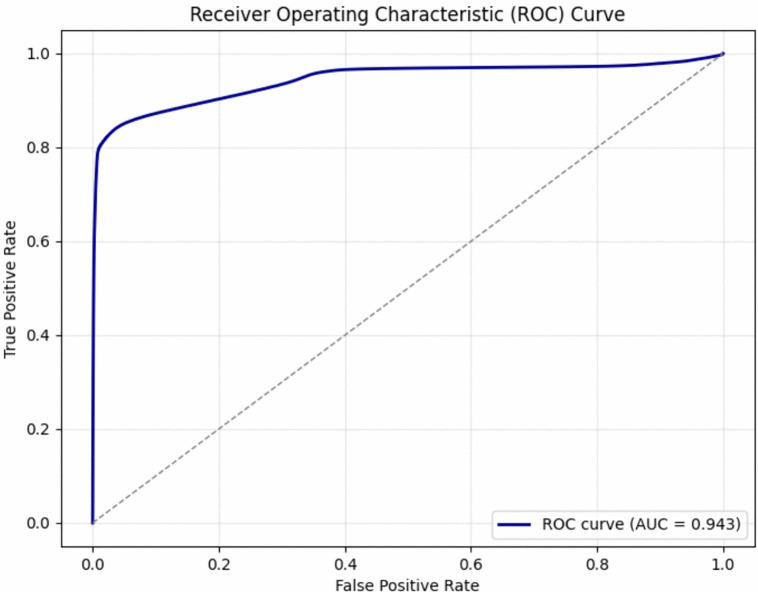
ROC curve.

To assess the completeness of threshold-independent voxel classification, we also reported ROC curves. The horizontal axis represents the“False positive rate,” and the vertical axis represents the“True positive rate.”. In Fig 8 the dark blue ROC curve deviates significantly from the gray diagonal representing“Nondiscriminatory”(AUC = 0.5) and extends with strong convexity towards the upper left corner (0,1). This yields an AUC of 0.943, confirming that the model maintains a robust true positive rate while effectively minimizing the false positive rate for all thresholds. Ultimately, this ROC analysis, as a secondary validation, confirmed the superior discriminative ability of the model, supporting the high Dice and IoU scores obtained in challenging, highly imbalanced tumor areas.

### 4.7. Ablation study

To directly respond to the central claim of this work, the ablation study is interpreted through the distinction between modeling-level innovations and supporting integrations. The three most important removals correspond to the three conceptual contributions of MamNet-PT. Removing the Mamba-based state-space pathway tests Core Innovation 1 (selective SSM embedding) and causes a marked degradation in Dice score, confirming that efficient long-range reasoning is essential for anatomically coherent tumor segmentation. Removing the gating/attention-based interaction pathway tests Core Innovation 2 (gated bidirectional fusion) and leads to a substantial decline in both Dice and recall, demonstrating that global information alone is insufficient unless it can be adaptively reconciled with local boundary evidence. Removing the Bayesian uncertainty mechanism tests Core Innovation 3 (multi-level Bayesian uncertainty propagation) and reduces both segmentation quality and reliability, indicating that confidence-aware inference is functionally beneficial rather than merely interpretive.

By contrast, the removal of pyramid fusion, Transformer-assisted decoding, adaptive loss, curriculum learning, or deep supervision should be interpreted as ablations of supporting integrations. These modules strengthen scale-awareness, optimization stability, and feature refinement, but they do not redefine the manuscript’s central conceptual claim. Their effect is therefore best understood as amplifying or stabilizing the three core innovations rather than replacing them.

All ablation experiments followed the same leakage-free evaluation protocol described in Section 4.1. Specifically, the patient-level data partition was kept fixed across all ablation settings, and slice extraction or patch sampling was performed only after each subject had been assigned to a single subset. Therefore, all performance differences in Table 6 reflect architectural or optimization changes under an identical subject-level evaluation setting, rather than changes in data splitting or slice-level information leakage.

**Table 6:** Patient-level ablation study under the fixed BraTS2020 train/validation/test partition.

Experimental Configuration	Accuracy (%)	F1(%)	Recall (%)	Dice (%)
Base(U-Net)	83.2 ± 0.6	85.5 ± 0.5	85.1 ± 0.6	81.4 ± 0.7
Full model: MamNet-PT(Ours)	98.7 ± 0.2	98.5 ± 0.2	98.1 ± 0.2	96.7 ± 0.3
-Full model w/o Pyramid Fusion	90.5 ± 0.4	87.1 ± 0.5	87.8 ± 0.5	86.2 ± 0.5
-Full model w/o Attention Mechanism	91.2 ± 0.4	90.6 ± 0.4	91.1 ± 0.4	87.4 ± 0.5
-Full model w/o Transformer	92.3 ± 0.3	89.4 ± 0.4	90.7 ± 0.4	89.6 ± 0.4
-Full model w/o Adaptive Loss	91.8 ± 0.4	91.3 ± 0.4	90.2 ± 0.5	89.9 ± 0.4
-Full model w/o Curriculum Learning	93.4 ± 0.3	93.1 ± 0.3	90.7 ± 0.4	90.6 ± 0.4
-Full model w/o Uncertainty Estimation	93.8 ± 0.3	91.7 ± 0.4	92.5 ± 0.4	91.1 ± 0.4
-Full model w/o Deep Supervision	94.6 ± 0.3	92.9 ± 0.3	93.4 ± 0.3	92.2 ± 0.4
-Full model w/o Mamba	92.7 ± 0.4	91.8 ± 0.4	92.1 ± 0.4	90.4 ± 0.5

The multi-scale pyramid fusion module contributes to task-specific scale-aware representation. By processing feature maps over multiple receptive-field ranges, it reduces two recurrent failure modes in brain tumor MRI: omission of small enhancing foci and discontinuity along diffuse edema boundaries. When this module is removed, the Dice score drops from 96.7% to 86.2%, representing the largest degradation among the supporting integrations. This result indicates that multi-resolution aggregation materially improves size-aware lesion representation and stabilizes contour delineation under substantial intra-tumoral heterogeneity.

The attention-gated refinement mechanism is designed to suppress irrelevant background responses and preserve lesion-relevant local evidence during fusion. Its practical effect is clear in the ablation study: removing the attention mechanism reduces accuracy from 98.7% to 91.2%, recall from 98.1% to 91.1%, and Dice from 96.7% to 87.4%. Qualitatively, the degradation is most obvious at low-contrast interfaces between tumor core, edema, and surrounding tissue, where false positives and boundary leakage become more frequent. These findings show that the gating mechanism improves lesion selectivity and boundary precision through input-dependent feature weighting.

The introduction of the Transformer module aims to address the limitations of traditional convolutional neural networks in modeling long-range dependencies. In the fourth experiment, we removed the Transformer decoder component and replaced it with a standard convolutional bottleneck structure. Results clearly show a significant decline in model performance for large tumor segmentation tasks requiring global contextual information. The Dice score decreased from 0.967 to 0.896, with segmentation continuity particularly affected in large tumors spanning multiple brain regions. In-depth analysis of feature response maps revealed that models lacking the Transformer module exhibited deficiencies in maintaining global consistency, producing more fragmented segmentation results. This finding validates our hypothesis: in brain tumor segmentation, the collaborative modeling of local features and global context is crucial for obtaining anatomically plausible segmentation results.

The adaptive multi-loss function combination mechanism balances the training contributions of different loss terms through dynamic weight adjustment. In the fifth experiment, we replaced the adaptive mechanism with a loss function using fixed weight ratios: Dice:0.6, BCE:0.3, Focal:0.1. Results showed that the fixed-weight strategy exhibited poorer training stability, with noticeable performance fluctuations emerging during the mid-training phase. The final model achieved a Dice coefficient of 0.899 on the validation set, representing a 6.8% decrease compared to the adaptive loss mechanism’s 0.967. The adaptive mechanism dynamically adjusts the weights of each loss term throughout training,prioritizing boundary optimization in the early stages and focusing on regional consistency in later stages. This dynamic adjustment strategy significantly enhances the model’s convergence and final performance.

The curriculum learning strategy implements a difficulty-aware sample-scheduling scheme in which structurally clear and high-contrast cases are emphasized during early training, followed by the progressive introduction of cases with blurred boundaries, low contrast, and complex morphology. In the sixth experiment, we replaced curriculum learning with traditional random sampling. Results indicate that models without curriculum learning converge more slowly during the early training stage and are more prone to suboptimal solutions. This decline is particularly evident in difficult samples, such as tumors with blurred boundaries and low contrast. Quantitative analysis shows that the F1-score on the challenging sample subset decreases from 0.985 to 0.931, corresponding to a 5.4% reduction. These results confirm that difficulty-aware sample scheduling improves optimization stability and generalization to complex cases.

The Bayesian uncertainty estimation module provides auxiliary confidence estimates for segmentation outputs through Monte Carlo Dropout. In the seventh experiment, we removed the uncertainty estimation component and retained only deterministic outputs. The uncertainty maps helped identify regions where the model produced less stable predictions, particularly low-contrast boundaries and small enhancing components. Further analysis showed an association between high-uncertainty regions and segmentation errors, supporting the usefulness of uncertainty estimation for retrospective model interpretation and failure analysis.

The deep supervision mechanism alleviates the vanishing gradient problem and promotes multi-level feature learning by introducing auxiliary supervision signals at different encoder depths. In the eighth experiment, we removed all deep supervision branches, retaining only the final output supervision signal. Results showed that models lacking deep supervision converged significantly slower, requiring more training epochs to achieve comparable performance. Within limited training cycles, the Dice coefficient of the non-depth-supervised model reached 0.922, 4.5% lower than the complete model. Feature visualization analysis indicates that the deep supervision mechanism promotes the learning of more discriminative feature representations across encoder layers, with intermediate layers demonstrating superior performance in tumor texture feature extraction.

The introduction of the Mamba module is based on recent advancements in modern state space models, aiming to address core challenges in long sequence modeling and global context capture. The ninth experiment conducted an in-depth analysis specifically targeting the Mamba module, evaluating its unique contribution to brain tumor segmentation by replacing it with a standard convolutional bottleneck layer. Results demonstrated that removing the Mamba module caused a significant decline in model performance, with the Dice coefficient dropping from the baseline 0.967 to 0.904,a 6.3% decrease statistically significant at p < 0.01. Further analysis revealed that the absence of the Mamba module systematically impaired the model’s ability to handle complex tumor morphologies, particularly in segmenting irregular boundaries and identifying diffuse tumor regions. From a computational perspective, the Mamba module efficiently models long-range dependencies in the image space through its selective state space mechanism. This capability, which traditional convolutional neural networks often approximate by stacking numerous layers, is achieved by Mamba with lower computational complexity while delivering comparable global contextual modeling capacity.

### 4.8. Integrated interpretation of results

Taken together, the results reveal a coherent performance story. MamNet-PT is strongest when brain tumor segmentation requires both global continuity and local discrimination. The overall gains over state-of-the-art baselines (Table 3), the favorable efficiency-complexity trade-off (Table 4), and the strong WT/TC/ET Dice scores (Table 5) jointly show that the model captures anatomically plausible tumor extent without sacrificing fine-grained lesion evidence. This pattern is consistent with the proposed architecture: Mamba-based state-space modeling contributes efficient global reasoning, while pyramid fusion and gated local refinement preserve multi-scale boundary and small-lesion cues.

The residual errors are equally informative. The failure cases show that the model can still underestimate very small enhancing foci, fragmented satellite lesions, or extremely low-contrast infiltrative margins. These cases are not outliers unrelated to the main findings; they define the current boundary of the method. The ablation study explains why these errors persist at the hardest end of the distribution: removing Mamba weakens global continuity, removing pyramid fusion or attention-guided refinement reduces sensitivity to small and ambiguous structures, and removing uncertainty estimation obscures where predictions should be interpreted cautiously. Therefore, the empirical picture is internally consistent: MamNet-PT substantially reduces the global-local trade-off relative to prior methods, but it does not eliminate the most difficult cases created by extreme lesion size, weak contrast, and diffuse tumor infiltration. This critical assessment more precisely defines both the capability and the remaining challenge of the proposed method.

## 5. Discussion

### 5.1. Existing problems

Despite the strong performance achieved by MamNet-PT on the BraTS2020 benchmark, several limitations should be acknowledged. First, the present evaluation was conducted on a retrospective public benchmark with standardized preprocessing and curated annotations. Although BraTS2020 provides a valuable and widely used platform for reproducible comparison, it cannot fully represent the heterogeneity of external MRI data acquired across different institutions, scanner vendors, magnetic field strengths, acquisition protocols, contrast-administration settings, and preprocessing pipelines. Therefore, the reported performance should be interpreted as evidence of effectiveness under a retrospective benchmark setting rather than as proof of cross-domain generalizability. External validation on independent multi-institutional and multi-scanner cohorts remains necessary before stronger conclusions can be drawn regarding robustness in heterogeneous imaging environments.

Second, although the proposed architecture improves the interaction between global contextual modeling and local boundary refinement, it does not eliminate all difficult segmentation cases. Extremely small enhancing tumor regions, low-contrast infiltrative margins, fragmented satellite lesions, and highly irregular tumor morphologies remain challenging. These residual errors are particularly important because they occur in regions where MRI appearance is inherently ambiguous and where foreground voxels may occupy only a small proportion of the image. The qualitative failure cases and ablation results indicate that Mamba-based state-space modeling, pyramid fusion, and attention-guided refinement reduce these errors but do not completely resolve the limitations imposed by extreme scale variability, weak contrast, and discontinuous lesion distribution. Future work should therefore investigate more contrast-sensitive feature representations, stronger small-lesion supervision, and more systematic subgroup-level failure analysis.

Third, the uncertainty maps generated by Monte Carlo Dropout were evaluated only as auxiliary research outputs for retrospective confidence characterization and failure-mode analysis. The present study did not assess probability calibration under domain shift, expert reader performance, annotation efficiency, treatment-planning impact, workflow integration, or patient-level outcomes. Consequently, these uncertainty estimates should not be interpreted as clinically validated indicators of diagnostic reliability or decision-making benefit. Future studies should incorporate reliability diagrams, expected calibration error, external validation cohorts, and subgroup-specific calibration analysis to determine whether uncertainty-aware outputs remain reliable under heterogeneous imaging conditions.

Fourth, the current implementation relies on 2D slice-based training and inference after patient-level partitioning. This design improves computational efficiency and helps maintain a leakage-free evaluation protocol, but it may not fully exploit through-plane anatomical continuity in 3D multimodal MRI volumes. Tumor components extending across adjacent slices, especially diffuse edema or small discontinuous enhancing foci, may benefit from explicit volumetric modeling. Future extensions could explore 2.5D or fully 3D state-space architectures, volumetric uncertainty estimation, and cross-slice consistency constraints while maintaining feasible memory consumption.

Finally, although MamNet-PT shows favorable computational efficiency under the experimental hardware configuration, inference speed and memory usage alone do not establish deployment readiness. Practical use in external settings would require independent validation, standardized preprocessing protocols, robustness testing under acquisition variability, prospective reader studies, and formal workflow evaluation. Therefore, MamNet-PT should be positioned as a retrospective segmentation research framework that advances global-local feature interaction and uncertainty-aware analysis, rather than as a clinically validated decision-support system.

### 5.2. Interpretation of model positioning and uncertainty estimation

MamNet-PT should be positioned not as a heterogeneous accumulation of contemporary modules, but as a hypothesis-driven solution to a fundamental problem in medical image segmentation: how to achieve globally coherent yet locally precise delineation under limited annotated data and substantial tumor heterogeneity. From this perspective, the contribution of the model resides primarily in three coordinated modeling-level innovations rather than in the mere coexistence of CNN, Transformer, and Mamba components.

The first innovation, selective SSM embedding, addresses the need for long-range anatomical reasoning without incurring the quadratic cost of full self-attention. The second innovation, gated bidirectional fusion, ensures that global contextual information does not erase or oversmooth lesion boundaries, but is adaptively reconciled with boundary-sensitive local evidence. The third innovation, multi-level Bayesian uncertainty propagation, exposes regions where predictions remain unstable under stochastic inference. Together, these mechanisms provide a coherent methodological framework for improving retrospective brain tumor segmentation performance.

The uncertainty maps generated by MamNet-PT should be interpreted as auxiliary research outputs for confidence characterization and failure-mode analysis. They were not evaluated in PACS environments, multidisciplinary tumor-board settings, surgical planning, radiotherapy planning, treatment-response monitoring, or patient-care workflows. Therefore, no claim is made that these uncertainty maps improve clinical decision-making, workflow efficiency, or patient outcomes. Their role in this study is limited to identifying regions of prediction instability within the retrospective benchmark evaluation.

From a methodological perspective, the combination of segmentation probability maps and predictive variance maps provides a more informative research output than deterministic segmentation alone. However, further validation is required to determine whether uncertainty-aware outputs are reliable under domain shift, whether they are calibrated across institutions and scanners, and whether they provide measurable benefit to expert readers.

### 5.3. Future improvements

Although MamNet-PT demonstrates strong performance on the BraTS2020 benchmark, several important directions remain for future work. First, external validation on independent multi-institutional and multi-scanner cohorts is necessary to assess robustness under domain shift. Differences in imaging protocols, scanner vendors, acquisition parameters, contrast administration, and patient populations may affect segmentation performance and uncertainty estimation. Therefore, future studies should evaluate MamNet-PT on external datasets before drawing conclusions about generalizability.

Second, prospective reader studies are needed to determine whether the segmentation outputs and uncertainty maps provide measurable benefit to human experts. Such studies should evaluate reader accuracy, annotation variability, interpretation time, confidence, and failure recognition under controlled conditions. Without this evidence, the model should be regarded as a retrospective segmentation framework rather than a clinically validated decision-support system.

Third, future research should examine calibration and reliability under distribution shift. Although Monte Carlo Dropout provides a practical approximation of predictive uncertainty, uncertainty estimates may become miscalibrated when applied to images from unseen institutions or acquisition protocols. Reliability diagrams, expected calibration error, external test cohorts, and subgroup analyses should be incorporated into future evaluation protocols.

Finally, computational efficiency can be further improved by exploring lightweight state-space modules, dynamic inference strategies, and model compression. However, claims regarding real-time use or workflow integration should be made only after dedicated latency testing, safety analysis, and workflow evaluation.

## 6. Conclusion

This study presents MamNet-PT, a Mamba-enhanced hybrid architecture for uncertainty-aware brain tumor segmentation from MRI. The proposed framework was designed to address a central challenge in brain tumor segmentation: achieving globally coherent lesion representation while preserving fine-grained boundary details under substantial tumor heterogeneity, severe foreground-background imbalance, and limited annotated data. By integrating selective state-space modeling, gated global-local feature fusion, multi-resolution pyramid aggregation, and Monte Carlo Dropout-based uncertainty estimation, MamNet-PT provides a unified architecture for accurate and confidence-aware retrospective segmentation.

The experimental results on the BraTS2020 benchmark demonstrate the effectiveness of the proposed design. MamNet-PT achieved an overall Dice score of 96.7%, an IoU of 95.4%, an F1-score of 98.5%, and a Recall of 98.1% on the independent testing cohort. Subregion-level analysis further showed strong segmentation performance for enhancing tumor, tumor core, and whole tumor regions, indicating that the model can simultaneously capture small enhancing foci, heterogeneous core structures, and spatially extended edema within the benchmark setting. These results support the architectural rationale that selective state-space modeling improves long-range contextual coherence, whereas gated local refinement and pyramid fusion enhance boundary sensitivity and scale-aware representation.

Ablation experiments further confirmed that the performance gains of MamNet-PT arise from the complementary contributions of its core components rather than from simple module stacking. The Mamba-based state-space pathway strengthens efficient long-range dependency modeling, the gated interaction mechanism adaptively balances global and local features, and the pyramid fusion module improves representation across tumor scales. In addition, Monte Carlo Dropout-based uncertainty estimation provides voxel-wise predictive variance maps that can be used for retrospective confidence characterization and failure-mode analysis, particularly in ambiguous regions such as low-contrast boundaries and small enhancing lesions.

Overall, MamNet-PT provides a robust and uncertainty-aware research framework for brain tumor segmentation. Its integration of selective state-space modeling, gated global-local fusion, multi-resolution feature aggregation, and uncertainty estimation offers a promising direction for developing segmentation models that are not only accurate under retrospective benchmark evaluation, but also more transparent in characterizing prediction confidence and remaining failure modes.
